# Yttrium Iron Garnet Thin Films with Very Low Damping Obtained by Recrystallization of Amorphous Material

**DOI:** 10.1038/srep20827

**Published:** 2016-02-10

**Authors:** Christoph Hauser, Tim Richter, Nico Homonnay, Christian Eisenschmidt, Mohammad Qaid, Hakan Deniz, Dietrich Hesse, Maciej Sawicki, Stefan G. Ebbinghaus, Georg Schmidt

**Affiliations:** 1 Institut für Physik, Martin-Luther University Halle-Wittenberg, Halle, 06120, Germany; 2 Max-Planck-Institut für Mikrostrukturphysik, Halle, 06120, Germany; 3 Institute of Physics, Polish Academy of Sciences, Al. Lotnikow 32/46, 02-668 Warszawa, Poland; 4 Institut für Chemie, Martin-Luther University Halle-Wittenberg, Halle, 06120, Germany; 5 Interdisziplinäres Zentrum für Materialwissenschaften, Martin-Luther University Halle-Wittenberg, Nanotechnikum Weinberg, Halle, 06120, Germany

## Abstract

We have investigated recrystallization of amorphous Yttrium Iron Garnet (YIG) by annealing in oxygen atmosphere. Our findings show that well below the melting temperature the material transforms into a fully epitaxial layer with exceptional quality, both structural and magnetic. In ferromagnetic resonance (FMR) ultra low damping and extremely narrow linewidth can be observed. For a 56 nm thick layer a damping constant of α = (6.15 ± 1.50) · 10^−5^ is found and the linewidth at 9.6 GHz is as small as 1.30 ± 0.05 Oe which are the lowest values for PLD grown thin films reported so far. Even for a 20 nm thick layer a damping constant of *α* = (7.35 ± 1.40) · 10^−5^ is found which is the lowest value for ultrathin films published so far. The FMR linewidth in this case is 3.49 ± 0.10 Oe at 9.6 GHz. Our results not only present a method of depositing thin film YIG of unprecedented quality but also open up new options for the fabrication of thin film complex oxides or even other crystalline materials.

YIG can be considered the most prominent material in spin dynamics in thin films and related areas. It is widely used in ferromagnetic resonance experiments^1^,^2^,^3^,^4^,^5^,^6^, research on magnonics^7^,^8^,^9^,^10^,^11^,^12^,^13^,^14^ and magnon-based Bose-Einstein-condesates^15^,^16^,^17^,^18^ because of its exceptionally low damping even in thin films. In research on spin pumping^19^,^20^,^21^,^22^,^23^ and investigation of the inverse spin hall effect^1^,^19^,^24^,^25^,^26^,^27^,^28^ it greatly facilitates experiments because it is an insulating material which avoids numerous side effects which occur when ferromagnetic metals are used^29^,^30^. The field of spin caloritronics^31^,^32^,^33^,^34^,^35^,^36^,^37^,^38^,^39^ also would not have developed that rapidly without the availability of a non-conducting magnet with long magnon lifetimes.

The new fields of applications have resulted in a growing need of high quality thin films, for example for integrated magnonics where layers need to be as thin as 100 nm or even less. While formerly only micrometer thick films were used which can be obtained by liquid phase epitaxy with very high quality^40^,^41^,^42^,^43^ ultrathin films are nowadays mostly fabricated by pulsed laser deposition (PLD) of epitaxial films at elevated temperature. Especially for ultra thin films (20 nm or less) grown by PLD quality is high but limited and best results so far show a linewidth in FMR of 2.1 Oe at 9.6 GHz^1^.

## Sample Fabrication

The amorphous YIG layers are deposited on (111) oriented gallium gadolinium garnet (GGG) substrates (CrysTec GmbH). The GGG substrate (10 × 5 mm^2^) is cleaned in acetone and subsequently in isopropanole both using ultrasonic agitation.

The substrate is fixed on the sample holder with conducting silver glue. The sample holder is baked out at 250 °C for 30 minutes on a hotplate.

For the deposition the sample holder is introduced into the PLD-chamber (TSST, background pressure 10^−9^ mbar). During the deposition 0.025 mbar of oxygen are used. The Laser (Coherent, COMPEX Pro 205) has a wavelength of 248 nm, and is operated at a fluency of 2.5 J cm^−2^ and a frequency of 5 Hz resulting in a growth rate of 0.5 nm min^−1^. After deposition the sample is cut into five samples of 2 × 5 mm^2^ which are then annealed in a quartz oven. Annealing is done in a pure oxygen atmosphere (99.997%) at ambient pressure at 800 °C for 30 minutes (sample A, 56 nm thick), at 800 °C for three hours (sample B, 20 nm thick), and at 900 °C for four hours (sample C, 113 nm thick). A number of annealing times and temperatures have been investigated, however, the lowest damping is observed for a temperature range of 800 °C to 900 °C and annealing times between 0.5 and 4 hours. Within these limits no systematic but only statistical variation of the damping and linewidth are observed. After annealing the samples are subject to various structural and magnetic characterization experiments.

## Structural characterization

Structural characterization is done by X-ray diffraction, X-ray reflectometry, transmission electron microscopy and Reflection high energy electron diffraction (RHEED).

## X-ray characterization. 

X-ray diffraction is performed by doing an *ω*/2*θ* scan of the (444) reflex and a rocking curve of the YIG layer peak.

**Figure 1:**
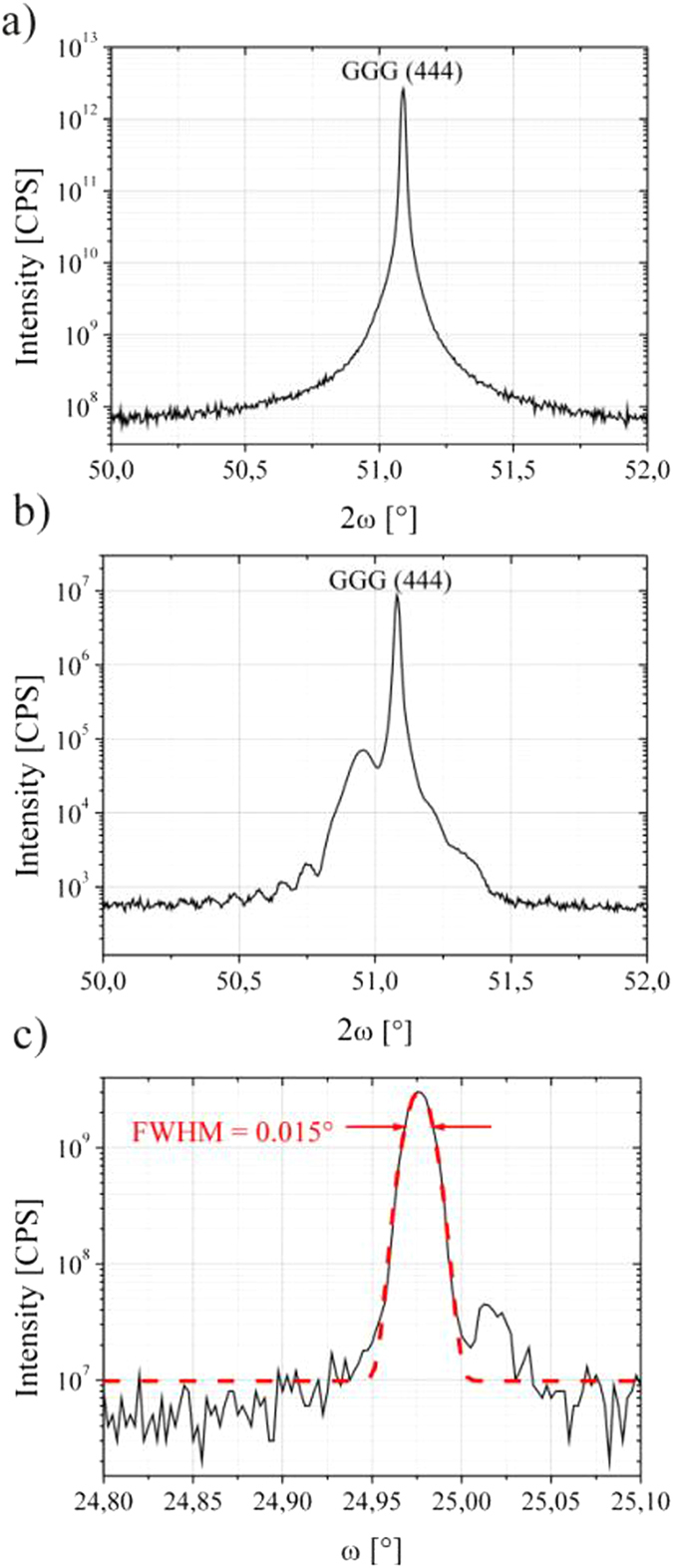
X-ray diffraction (*ω/2θ* scans) for an unannealed (**a**) and an annealed (**b**) YIG layer. Before annealing only the substrate peak is visible. After annealing the YIG peak clearly shows up. The position of the peak and the thickness fringes indicate fully pseudomorphic growth and smooth interfaces. (**c**) shows a rocking curve of the layer peak shown in (**b**). The full width at half maximum is only 0.015°. The dotted line shows a Gaussian fit to the peak.

**Figure 2:**
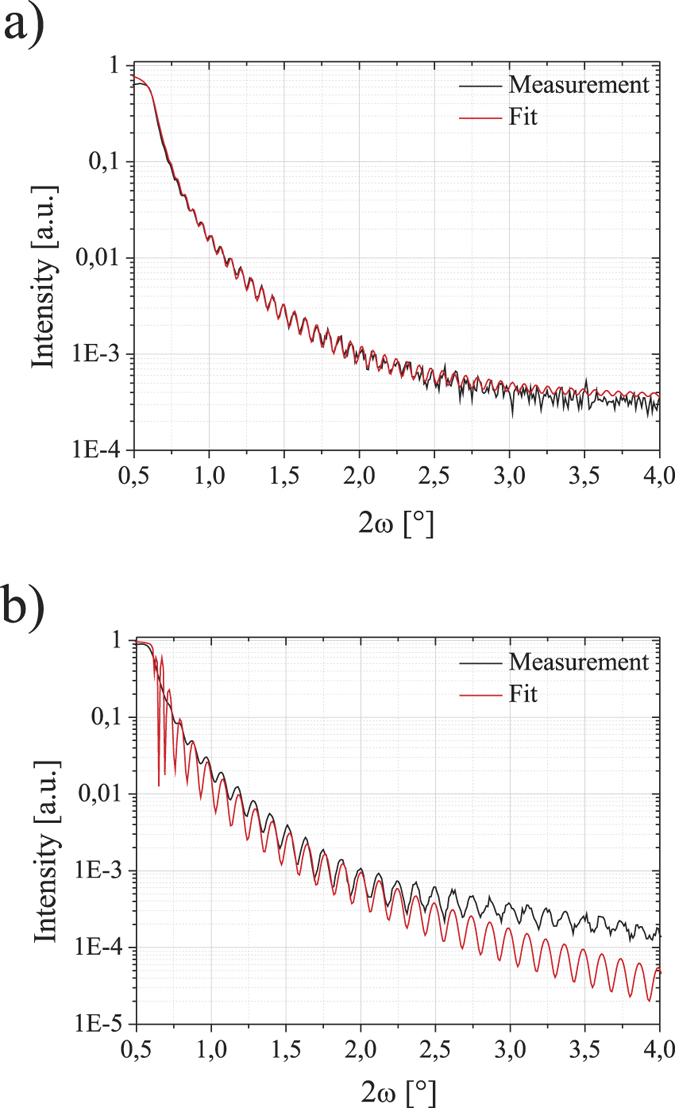
X-ray reflectometry (XRR) for sample C (**a**) and an unnanealed YIG layer (**b**). The red line is a fit coressponding to the measurement (black). For sample C the observed roughness is 0.2 nm. For the unnanealed sample the observed roughness is even lower then 0.1 nm.

Before annealing the diffraction pattern (Fig. 1a) only shows the peak of the GGG substrate indicating an amorphous or at least highly polycrystalline YIG film. A truly amorphous nature is confirmed by transmission electron microscopy as described below. After annealing, the diffraction pattern is completely changed. Figure 1b shows the *ω*/2*θ* scan for sample C. Here we clearly observe the diffraction peak of the YIG film at an angle corresponding to the small lattice mismatch of YIG on GGG which is only 0.057%. Even thickness fringes can be observed indicating a very smooth layer with low interface and surface roughness. From the peak positions we can deduce that all YIG layers are fully strained as is to be expected from the very small lattice mismatch. The layer peak is further investigated in a rocking curve (Fig. 1c) which shows a full width at half maximum (FWHM) of 0.015° indicating a fully pseudomorphic YIG layer. Roughness is also crosschecked using X-ray reflectometry (Fig. 2a) showing an RMS value of less than 0.2 nm^44^,^45^. It should, however, be noted that for not-annealed layers (Fig. 2b) the RMS roughness is even smaller than 0.1 nm.

## Transmission electron microscopy. 

**Figure 3:**
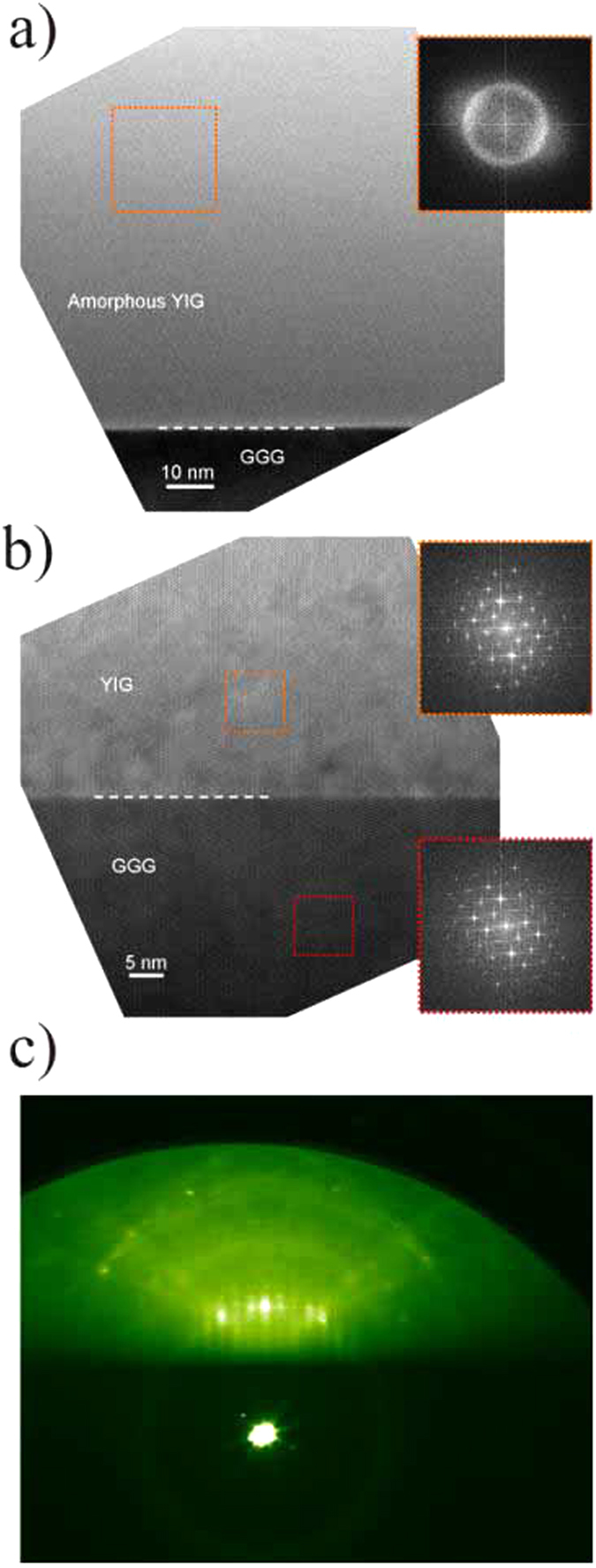
(**a**) A high resolution TEM (HRTEM) image of an amorphous YIG film on GGG substrate. The inset shows a FFT pattern from the region of interest (dotted frame) in the amorphous layer. (**b**) A HRTEM image of the interface between the annealed YIG film and the GGG substrate of sample C. The insets show FFT patterns from the regions of interest in the film and the substrate. The YIG film exhibits epitaxial growth with respect to the substrate and appears monocrystalline. (**c**) RHEED image obtained from the surface of an annealed YIG film (sample B). Kikuchi lines^46^ indicate a two dimensional highly ordered surface.

Transmission electron microscopy (TEM) is done on samples oriented for cross sectional view along the cubic crystalline axis. For the nominally amorphous sample the pictures (Fig. 3a) show a pure film without inclusions but also without any trace of polycristallinity. Further analysis using fast fourier transform confirms that the YIG layer is indeed completely amorphous.

For an annealed sample (sample C) the result of the TEM investigation is surprising (Fig. 3b). The sample is not only monocrystalline but it also shows no sign of inclusions or defects and even the interface to the GGG appears flawless.

## Reflection high energy electron diffraction. 

The atomic order of the layer surface after annealing is further investigated by Reflection high energy electron diffraction (RHEED). For this purpose sample B is again introduced into the PLD chamber after the annealing process. We use a differentially pumped RHEED-Gun from STAIB INSTRUMENTS which is operated at a Voltage of 30 kV. After evacuation a clear RHEED pattern is observed. The RHEED image (Fig. 3c) not only shows the typical pattern for a YIG surface during high temperature growth but also exhibits the so called Kikuchi lines^46^. We do not observe these lines in high temperature growth of epitaxial YIG. They are typically a sign of a surface of excellent two dimensional growth, again indicating that the crystalline quality of the annealed layers is extremely high.

## Magnetic characterization

Magnetic characterization is done using SQUID magnetometry and FMR at room temperature.

## SQUID magnetometry. 

**Figure 4:**
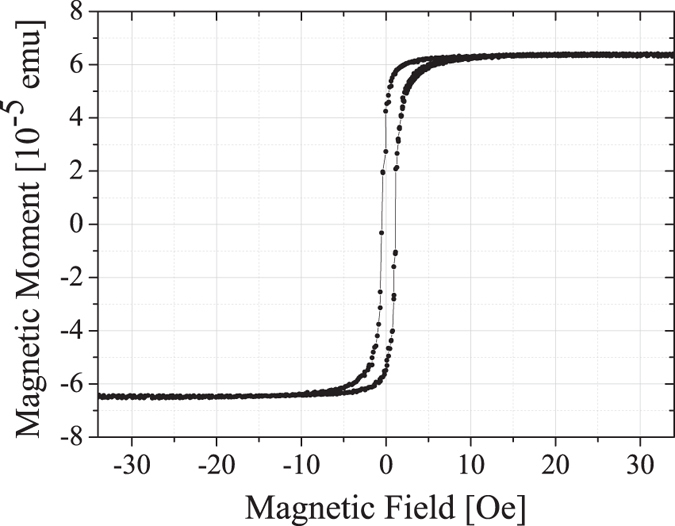
Hysteresis loop as measured by SQUID magnetometry for a 113 nm thick YIG sample after annealing. The paramagnetic background caused by the GGG substrate was subtracted.

In SQUID magnetometry hysteresis loops are taken on sample C. The data is corrected by subtracting a linear paramagnetic contribution which is caused by the GGG substrate. After correction the observed saturation magnetization is (115 ± 3) emu cm^−3^ which is approx. 20% below the bulk value^47^ (Fig. 4). The coercive field is determined as (0.8 ± 0.1) Oe. For sample A and B we were able to measure a saturation magnetization of (122 ± 3) emu cm^−3^ and (104 ± 3) emu cm^−3^. These values are approx. 15% and 27% below the bulk value, respectively. As we will show in the following section, these magnetization values correspond nicely to those obtained from FMR measurements.

## Ferromagnetic resonance. 

FMR is performed by putting the samples face down on a coplanar waveguide whose magnetic radio frequency (RF) field is used for excitation. The setup is placed in a homogenous external magnetic field which is superimposed with a small low frequency modulation. RF absorption is measured using a lock-in amplifier.

**Figure 5:**
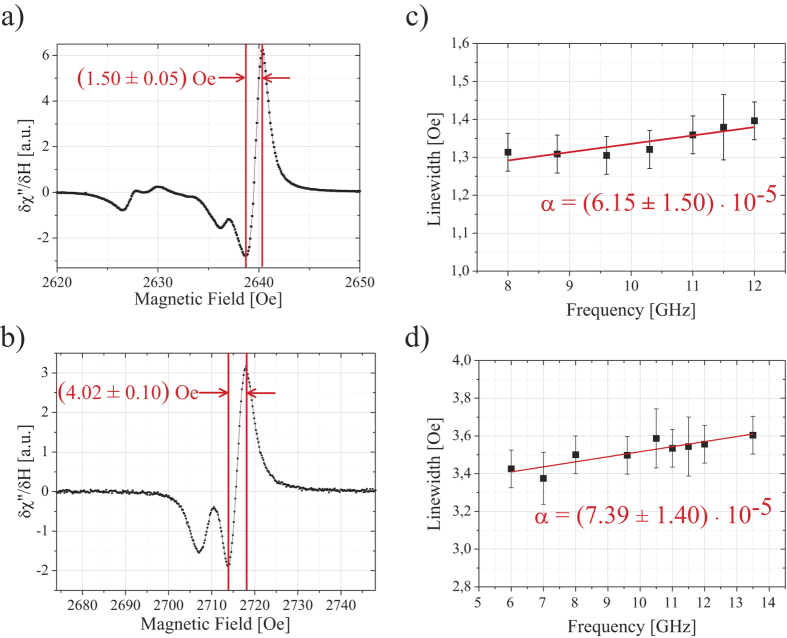
(**a**,**b**) FMR data obtained at 9.6 GHz for a 56 nm thick ((**a**), sample A) and a 20 nm thick ((**b**), sample B) YIG layer after annealing. The main resonance lines have a peak-to-peak linewidth of 1.50 ± 0.05 Oe (sample A) and 4.02 ± 0.10 Oe (sample B). This peak-to-peak linewidth corresponds to a true linewidth of 1.30 ± 0.05 Oe and 3.49 ± 0.10 Oe, respectively. (**c**,**d**) Frequency dependence of the FMR linewidth for sample A and sample B. The fits are a straight line corresponding to a damping of *α* = (6.15 ± 1.50) · 10^−5^ ((**c**), sample A) and *α* = (7.39 ± 1.40) · 10^−5^ ((**d**), sample B).

As expected no signal can be detected for unannealed YIG layers. For annealed samples a clear resonance is observed. Figure 5a shows the resonance signal for sample A. The linewidth which is obtained by multiplying the peak to peak linewidth of the derivative of the absorption by a factor of 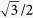
2,3,48,49 is only 1.30 ± 0.05 Oe at 9.6 GHz which is the smallest value for thin films reported so far^1^. In Fig. 5b the resonance of sample B is shown. Here the linewidth at 9.6 GHz is 3.49 ± 0.10 Oe. The additional peaks in Fig. 5a,b correspond to standing spin wave modes which are visible due to the extremely low damping in the layers. This is further confirmed by the change in line position with frequency which differs from the line shift of the uniform mode. The difference in resonance field (2639 Oe vs 2716 Oe) results from the different respective saturation magnetizations and different crystalline anisotropy which is evaluated below. For sample C the linewidth is 1.65 ± 0.10 Oe at 9.6 GHz (no figure).

In order to determine the damping constant *α* frequency dependent measurements are performed on sample A. The excitation frequency is varied between 8 and 12 GHz. Results are plotted in Fig. 5c. As described by Chang *et al.*^48^ and Liu *et al.*^2^ we first determine the gyromagnetic ratio *γ* using the Kittel equation for in-plane measurements


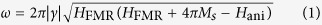


yielding *γ* = (2.80 ± 0.01) MHz Oe^−1^. The damping is then calculated from the frequency dependence of the linewidth to *α* = (6.15 ± 1.50) · 10^−5^. The linewidth at zero magnetic field is approx. 1.11 ± 0.05 Oe. This damping is even lower than the lowest value reported by Chang *et al.*^48^. It is interesting to note that Chang *et al.* did not observe a similarly small linewidth for their layer^48^. d’Allivy Kelly *et al.* on the other hand do observe a smaller linewidth for 20 nm thick layers of 2.1 Oe at 9.6 GHz^1^, however, the damping they find is three times as big as in our case. For sample B (20 nm) we found a gyromagnetic ratio of *γ* = (2.76 ± 0.01) MHz Oe^−1^ and a linewidth at zero magnetic field of approx. 3.25 ± 0.05 Oe and the damping was determined as *α* = (7.39 ± 1.40) · 10^−5^ which is also lower than any other value reported for similarly thin films (Fig. 5d). A systematic thickness dependence of the linewidth at zero magnetic field could not be observed for the samples investigated.

For comparison with the SQUID magnetometry results the saturation magnetization is determined by out-of-plane FMR measurements for sample A and B. From Kittel’s equation for out-of-plane measurements


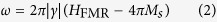


we find a saturation magnetization of 1520 ± 2 Oe (121 emu cm^−3^) for sample A and 1352 ± 2 Oe (107 emu cm^−3^) for sample B. These values fit very well with the observed saturation magnetization by SQUID magnetometry. We can thus conclude that the deviation from the literature value is not because of non-magnetic inclusions but because of an overall lowered saturation magnetization. The origin of this lower magnetization, however, cannot be determined at the moment. A possible explanation might be a deviation in stochiometry, especially in the oxygen content. In order to determine possible crystalline anisotropy we compare this value to the effecitve magnetization 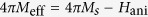 obtained from the in-plane FMR measurements. The respective values of 1795 ± 6 Oe for sample A and 1738 ± 28 Oe for sample B show that an out-of-plane anisotropy field 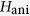 of −275 Oe for the 56 nm sample (sample A) and −386 Oe for the 20 nm sample (sample B) exist which is in good agreement with literature^50^.

## Discussion

In conclusion we can state that using high temperature annealing in oxygen atmosphere it is possible to transform amorphous YIG layers of tens of nanometers of thickness into epitaxial thin films with extremely small FMR linewidth and exceptionally low damping. The crystalline quality is extremely high. Our findings may thus present a new and easy route for thin film fabrication of epitaxial complex oxides.

## Methods

### X-ray characterization

The X-ray characterizations are done using a Bruker D8 diffractometer. CuK_*α*1_-radiation is focused on the sample through a 40 mm Göbel-mirror and a 2 bounce channel cut monochromator. The beam divergence of the primary beam is 0.007°. For the unlocked 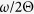-scan of the (444)-reflex a scintillation detector with a detector slit of 0.2 mm is used. To determine the mosaicity of the YIG layer the beam is centered on the layer peak, the detector with a 0.2 mm slit is kept on the 2Θ position and the sample is scanned in *ω* with a distance of 330 mm between sample and slit. For the XRR-experiments an additional knife edge stage with a slit of 300 *μ*m (knife edge) is used.

### Transmission electron microscopy

A FEI Nova Nanolab 600 focused ion beam equipment with Ga ions was employed to prepare ultra-thin cross-section lamellae for TEM observation. The lamellae were cut along one of the in-plane cubic axes of the GGG substrate. The FIB procedure was performed with an accelerating voltage of 30 kV at the beginning, and eventually it was dropped to 5 kV at the final stage of thinning. The ion beam current was about 50 pA. A final cleaning process was applied using a voltage of 2 kV and a current of 20 pA to remove redeposited material.

The high-resolution TEM (HRTEM) images were acquired in a JEOL JEM 4010 electron microscope equipped with a LaB_6_ electron gun at an accelerating voltage of 400 kV. The point resolution of the microscope is 1.6 Å and the lattice resolution is 1.0 Å. The images were obtained under parallel illumination of the electron beam using an objective aperture of 120 *μ*m to collect as many beams in the objective plane as possible for image formation. A GATAN Ultrascan (US 1000, 2k × 2k) CCD camera was used to record the images digitally. Fast Fourier Transforms were obtained using the GATAN DigitalMicrograph software. FFT patterns are 512 × 512 pixels in size.

### SQUID magnetometry

Most of the SQUID-measurements are done in a Quantum Design MPMS3 SQUID-Vibrating sample magnetometer. Before the measurement the magnet is reset to zero field. The hysteresis-loop is taken from −50 Oe to 50 Oe using sub-Oe field steps. The averaging time for each data point is 5 s. In addition a precise calibration with respect to sample geometry was done using a commercial MPMS-XL5 SQUID magnetometer strictly observing the recipes allowing precise magnetometry of thin layers on bulky substrates outlined by Sawicki *et al*.^51^.

### Ferromagnetic resonance

All FMR measurements are done at room temperature. The sample is put face down on a coplanar waveguide (copper, width of central conductor 600 *μ*m). The external magnetic field, which is generated by a rotatable electromagnet is aligned along the waveguide and along the long side of the sample (inplane geometry). The resolution of the field control is better than 0.05 Oe. The external field is modulated at a frequency of 184 Hz and an amplitude of less than 0.25 Oe, allowing for lock-in detection of the FMR signal. The microwave signal is applied to the waveguide using a RHODE&SCHWARZ, SMF 100A generator and the excitation power is kept in the range of −3 dBm to +10 dBm (excitation power was kept constant for each sample, respectively). Absorption is measured using a Schottky diode Whose signal is fed into a lock-in amplifier and then measured using a Agilent 34420A nanovoltmeter.

## Additional Information

**How to cite this article**: Hauser, C. *et al.* Yttrium Iron Garnet Thin Films with Very Low Damping Obtained by Recrystallization of Amorphous Material. *Sci. Rep.* **6**, 20827; doi: 10.1038/srep20827 (2016).
